# Mycotic keratitis caused by concurrent infections of exserohilum mcginnisii and candida parapsilosis

**DOI:** 10.1186/1471-2415-13-37

**Published:** 2013-08-01

**Authors:** Wen-Ya Qiu, Yu-Feng Yao

**Affiliations:** 1 Department of Ophthalmology, Sir Run Run Shaw Hospital, Zhejiang University School of Medicine, Zhejiang, P. R. China, 3 Qingchun Road East, Hangzhou 310016, China

**Keywords:** Mycotic keratitis, *Exserohilum mcginnisii*, *Candida parapsilosis*

## Abstract

**Background:**

Mycotic keratitis in human cornea has been rarely reported to be associated with a co-infection of filamentous fungi and yeast. This paper aims to report a case of mycotic keratitis concurrently infected by *Exserohilum mcginnisii* and *Candida parapsilosis*.

**Case presentation:**

A Chinese female presented two superposed corneal infiltrates with different size and texture on her left eye. In vivo confocal microscopy showed hyper-reflective multiple linear with highly branching structures distributing in the anterior corneal stroma. Inoculations of the corneal lesion scrape concurrently grew two similar superposed colonies on Sabouraud dextrose and chocolate agar plate. The larger colony exhibited mould, cottony and floccose at the edge, while the smaller one showed creamy and shiny surface. Modified slide culture for mould revealed hyphae were septate, and conidia were brown, smooth-walled, cylindrical to slight clavate with 6 to 13 pseudosepta. Based on the morphology of microscopic and macroscopic characteristics, the mould was identified as *Exserohilum mcginnisii.* Smear of the non-mould colony showed ellipse or ovoid budding yeast-like cells with abundant pseudomycelium. Vitek Yeast Biochemical Card test identified the yeast as *Candida parapsilosis.* With treatment of combined oral itraconazole with topical amphotericin B, a complete resolution of the corneal infiltrate was achieved within 1.5 months.

**Conclusion:**

This is the first documented case of human corneal infection by *Exserohilum mcginnisii*, and also the first report providing evidence of mycotic keratitis in human cornea concurrently infected by filamentous fungi and yeast.

## Background

Majority of the pathogens isolated from human cornea with keratomycosis are hyaline fungi such as *Fusarium, Aspergillus*[1]. The dematiaceous fungi such as *Alternaria, Curvularia, Exserohilum* are uncommon causes of keratomycosis [1]. The *Exserohilum* spp. is usually associated with infections in paranasal sinus, skin and subcutaneous tissue, and is very rarely reported to cause keratomycosis [2]. *Exserohilum mcginnisii* has not yet been isolated as pathogen causing human corneal phaeohyphomycosis. *Candida parapsilosis* is an opportunistic pathogen that may cause human mycotic keratitis. We report herein a case of mycotic keratitis presented two superposed corneal infiltrates where *Exserohilum mcginnisii* and *Candida parapsilosis* were cultured simultaneously in the same culture plate.

## Case presentation

**Figure 1:**
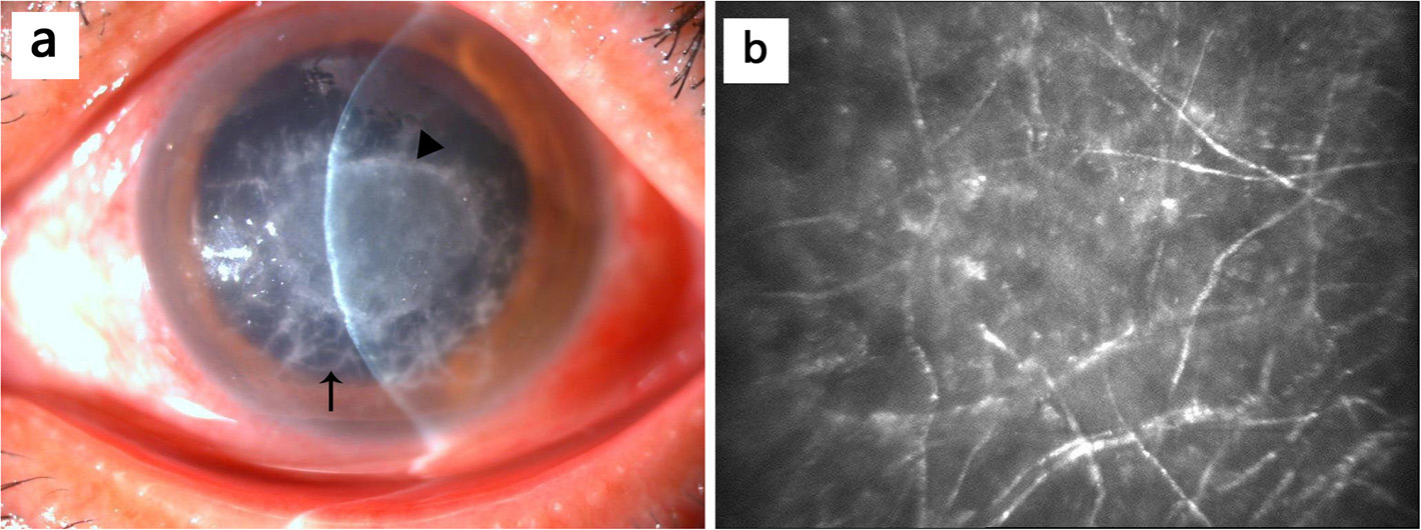
**Slit lamp photograph and confocal microscopy photograph at first visit. (a)** Slit lamp photograph showing two superposed corneal infiltrates in the patient cornea of the left eye. The larger infiltrate, involving anterior stroma of the cornea, approximate 8 mm in diameter, exhibiting gray interlaced braid-grid texture and irregular feathery margin (arrow). The smaller infiltrate superimposing on the larger one, characterized by slight elevation in oval shape and in gray color with rough surface and comparatively clear margin (triangle). **(b)** Confocal microscopy photograph showing multiple linear and highly branching and intersecting hyper-reflective structures spreading out and distributing mainly in the anterior stroma of the cornea. (original magnification ×1000).

A 49-year-old Chinese female felt foreign body sensation of her left eye on an occasion of weaving bamboo baskets. She visited the local ophthalmologist who prescribed her antibiotic eyedrops and intravenous Cefradine for suspicion of infectious keratitis. Nevertheless, she developed progressively exacerbating irritation, pain and remarkably decreased vision. Forty days later, the patient was referred to us. Initial examination exhibited two superposed corneal infiltrates. The larger infiltrate presented gray interlaced braid-grid texture and irregular feathery margin, mainly involving the anterior corneal stroma (Figure 1a). The smaller infiltrate, superimposing on the larger one, manifested slight elevation in oval shape and gray color with rough surface (Figure 1a). In vivo confocal microscopy showed multiple linear and highly branching and intersecting hyper-reflective structures distributing mainly in the anterior stroma of the cornea (Figure 1b). Visual acuity was finger counting OS. No abnormalities were found in the right eye with visual acuity 20/16.

Clinical diagnosis of fungal keratitis was made at the initial visit. Scraping of the corneal lesion was performed for fungal and bacterial cultures. The patient was given oral itraconazole 300 mg daily, topical 0.15% amphotericin B eyedrops every 30 min, together with 0.3% Oflaxacin eyedrops 4 times daily. Two weeks later, the interlaced braid-grid infiltration with its feathery margin of the larger infiltrate regressed remarkably in the stroma. The smaller infiltrate also dwindled in size significantly. Both the larger and the smaller infiltrates completely resolved one more month later, resulting in corneal scarring involving the optical axis. No recurrence observed over 2 years of follow-up. At her final visit, uncorrected visual acuity of the left eye was 20/40.

**Figure 2:**
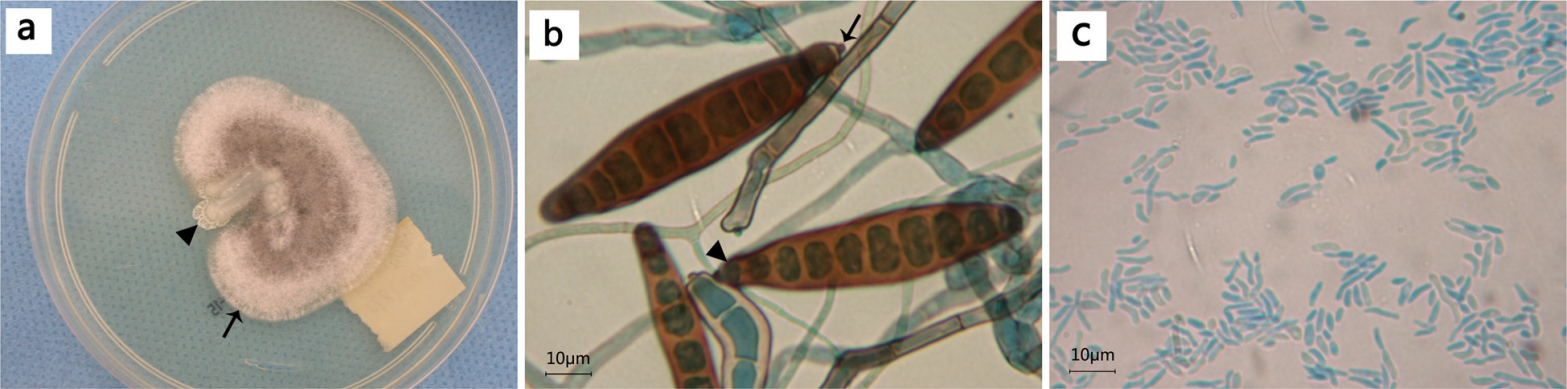
**Macroscopic and microscopic photographs of the mould and non-mould fungi. (a)** Seventy-two hours after culture of samples scraped from the patient cornea, two superposed colonies were observed growing on SDA. The larger colony (arrow) was mould, cottony and floccose at the edge with pale brown color, which was embedded by a smaller colony showing creamy and shiny appearance (triangle). **(b)** In slide culture of the mould colony, conidia are cylindrical to slight clavate with 6 to 13 pseudosepta. The hila of the conidia are black and distinctly protuberant (arrow). The pale end cells of the conidia are not separated from the intercalary golden-brown cells by thick-walled distosepta (triangle). (original magnification ×800). **(c)** In smearing of the non-mould colony, ellipse or ovoid budding yeast-like cells, 2.0-3.5 x 3.0-4.5 µm in size, and abundant pseudomycelium consisting of elongate cells, 2.0-3.5 × 10–15 μm in size are also observed. (original magnification ×800).

Inoculations of the corneal lesion samples concurrently grew two similar superposed colonies on SDA and chocolate agar plates, in which the larger colony presented molds like hairy and floccose towards the edge (Figure 2a), whereas the smaller one was yeast-like colony with creamy and shiny surface (Figure 2a). We used a modified slide culture, a technique developed by us and reported elsewhere previously to observe the microscopic characteristics of the mould colony [1]. Examination of modified slide culture revealed that hyphae were septate, subhyaline to pale to mid brown. The conidiophores were simple, erect, with somewhat flexouse apical part. The conidia were brown, smooth-walled, cylindrical to slight clavate, 60–100 x 10-15μm in size, with 6 to 13 pseudosepta (Figure 2b). The hila of the conidia were black and distinctly protuberant (Figure 2b). The pale end cells of the conidia were not separated from the intercalary golden-brown cells by thick-walled distosepta (Figure 2b). Based on the morphology of microscopic characteristics in the slide culture together with macroscopic feature growing in SDA, the mould colony was identified as *Exserohilum mcginnisii*[2-4]. The microscopic characteristics of the non-mould colonies after smearing in the slide showed ellipse or ovoid budding yeast-like cells, 2.0-3.5 × 3.0-4.5 μm in size, and abundant pseudomycelium consisting of elongate cells, 2.0-3.5 × 10–15 μm in size (Figure 2c). Furthermore, we used Vitek Yeast Biochemical Card (YBC; bioMérieux, Inc., Hazelwood, Mo.) through substrate assimilation assay to identify the non-mould colony in clinical microbiology laboratory of our hospital [5], and the results showed the isolate was *Candida parapsilosis*.

## Discussion and conclusion

To our knowledge, this is the first report providing evidence of a case of human keratomycosis infected by filamentous fungi and yeast. Clinically, there were two infiltrates superposing together in the cornea clearly showing significantly different clinical features, of which the larger one was in accordance with characteristics of filamentous fungal keratitis whereas the smaller one had appearance similar to yeast fungal keratitis. Based on clinical features (Figure 1a) and confocal microscopic findings (Figure 1b) of the cornea, clinical diagnosis of filamentous fungal keratitis can be established with no difficulty in our patient. Since dematiaceous fungus was subsequently isolated from the corneal lesion, diagnosis of filamentous fungal keratitis becomes definite. On the other hand, the characteristics of the smaller infiltrate clinically resembled yeast keratitis, coupled with two repeated isolations of the same pathogen growth of *candida parapsilosis* in cultures through corneal scrapings at different time intervals, which enabled to exclude the possibility of contamination in culture, making the diagnosis of yeast keratitis be also definite in this patient.

One of the two concurrent etiological pathogens isolated from this case was identified as *Exserohilum mcginnisii*. The genus *Exserohilum* shares somewhat similar morphology with genera *Bipolaris* and *Drechslera*, but they can be differentiated in further detail according to their microscopic morphologic characteristics. *Exserohilum* has cylindrical to slight clavate conidia with 6–13 pseudosepta, forming conidia with a strongly protruding truncate hilum [2-4]. But in *Drechslera* species, conidia have 2–3 distoseptate, and the hilum does not protrude. *Bipolaris* species has 4–5 distoseptate, and its hilum protrudes only slightly [4]. Among *Exserohilum* genus, there are three species: *longirostratum, rostratum* and *mcginnisii*. *Exserohilum longirostratum* has two types of conidia, a short and a long one. *Exserohilum rostratum* has unique characteristics of its conidia with darkly pigmented bands at the ends, which *Exserohilum mcginnisii* does not share. Corneal phaeohyphomycosis caused by *Exserohilum rostratum*[6-8], and *Exserohilum longirostratum*[9] has previously been described in several case reports. However, there is no report that *Exserohilum mcginnisii* was the pathogen in human keratomycosis. The case we report herein is the first documented case of human corneal infection by *Exserohilum mcginnisii*.

It is not rare in reports that *Candida parapsilosis* can cause endophthalmitis [10,11], whereas *Candida parapsilosis* causing corneal infection is not common. Literature review indicates the manifestations of *Candida parapsilosis* causing human mycotic keratitis vary greatly, including crystalline keratopathy, supportive keratitis, yellow-white infiltrate with dry raised slough and feathery edges, and severe necrotic stromal inflammation [12-15]. In our case, as compared with filamentous fungus of *Exserohilum mcginnisii* causing intra-stromal infiltration, *Candida parapsilosi* seems mainly grow in the superficial cornea, exhibiting comparatively dense and rough lesion with slight elevation on the corneal surface.

The isolated pathogens of *Exserohilum Mcginnisii* and *Candida parapsilosi* from the patient were both sensitive to itraconazole and amphoterycin B in vitro drug sensitivity test (data not shown), which was correlated well with the clinical result showing the corneal infiltrates responding well to the medication of oral itraconazole combined with topical amphoterycin B eyedrops in the patient.

To conclusion, this is the first documented case of human corneal infection by *Exserohilum mcginnisii*, and also the first report providing definite evidence of mycotic keratitis in human cornea concurrently infected by filamentous fungi and yeast.

## Informed consent

A written informed consent was obtained from the patient to publish this case report.

## Competing interests

The authors declare that they have no competing interests.

## Authors’ contributions

W-YQ: patient interaction and diagnosis, pathogen identification. Y-FY: draft the manuscript. Both authors read and approved the final manuscript.

## Pre-publication history

The pre-publication history for this paper can be accessed here:

http://www.biomedcentral.com/1471-2415/13/37/prepub
